# Web-Based Assessment of Mental Well-Being in Early Adolescence: A Reliability Study

**DOI:** 10.2196/jmir.5482

**Published:** 2016-06-15

**Authors:** Christoph Hamann, Frauke Schultze-Lutter, Leila Tarokh

**Affiliations:** ^1^ University Hospital of Child and Adolescent Psychiatry and Psychotherapy University of Bern Bern Switzerland; ^2^ Institute of Pharmacology and Toxicology University of Zurich Zurich Switzerland; ^3^ Psychiatry and Human Behavior The Alpert Medical School of Brown University Providence, RI United States

**Keywords:** early adolescence, online assessment, reliability

## Abstract

**Background:**

The ever-increasing use of the Internet among adolescents represents an emerging opportunity for researchers to gain access to larger samples, which can be queried over several years longitudinally. Among adolescents, young adolescents (ages 11 to 13 years) are of particular interest to clinicians as this is a transitional stage, during which depressive and anxiety symptoms often emerge. However, it remains unclear whether these youngest adolescents can accurately answer questions about their mental well-being using a Web-based platform.

**Objective:**

The aim of the study was to examine the accuracy of responses obtained from Web-based questionnaires by comparing Web-based with paper-and-pencil versions of depression and anxiety questionnaires.

**Methods:**

The primary outcome was the score on the depression and anxiety questionnaires under two conditions: (1) paper-and-pencil and (2) Web-based versions. Twenty-eight adolescents (aged 11-13 years, mean age 12.78 years and SD 0.78; 18 females, 64%) were randomly assigned to complete either the paper-and-pencil or the Web-based questionnaire first. Intraclass correlation coefficients (ICCs) were calculated to measure intrarater reliability. Intraclass correlation coefficients were calculated separately for depression (Children’s Depression Inventory, CDI) and anxiety (Spence Children’s Anxiety Scale, SCAS) questionnaires.

**Results:**

On average, it took participants 17 minutes (SD 6) to answer 116 questions online. Intraclass correlation coefficient analysis revealed high intrarater reliability when comparing Web-based with paper-and-pencil responses for both CDI (ICC=.88; *P*<.001) and the SCAS (ICC=.95; *P*<.001). According to published criteria, both of these values are in the “almost perfect” category indicating the highest degree of reliability.

**Conclusions:**

The results of the study show an excellent reliability of Web-based assessment in 11- to 13-year-old children as compared with the standard paper-pencil assessment. Furthermore, we found that Web-based assessments with young adolescents are highly feasible, with all enrolled participants completing the Web-based form. As early adolescence is a time of remarkable social and behavioral changes, these findings open up new avenues for researchers from diverse fields who are interested in studying large samples of young adolescents over time.

## Introduction

Adolescent development is long, complex, and highly individual (eg, [1-4]). In order to capture the dynamic process that is adolescent development, large sample sizes or longitudinal assessment is often required [5]. Access to large samples of adolescents over extended periods can be challenging because of factors such as changing of schools, moving, or unstable family environments [6]. Thus, data collection is often cumbersome, expensive, and can result in dropouts that may influence results [7,8].

One way to circumvent some of these difficulties is through the administration of Web-based questionnaires. Adolescents today have regular access to the Internet either at school or at home. For example, according to the Pew Research Center, 95% of teenagers (12-17 years) have Internet access in the United States [9], and on average a European teenager uses online media for 66 minutes per day [10]. The ever-increasing use of the Internet among adolescents represents an emerging opportunity for researchers to gain access to large samples, which can be queried over several years longitudinally. Subjects can complete such questionnaires wherever and whenever it suits them using different media, for example, tablets, laptops, or mobile phones, and researchers can monitor data quality instantaneously. Furthermore, the Internet offers diverse options for communication between participants and researchers, which can be used to bolster participation and to minimize dropouts [8].

Previous studies have shown that adolescents (13-20 years) [11,12] can accurately and reliably fill out Web-based questionnaires about their mental and physical well-being. However, few studies have addressed the reliability of Web-based questionnaires in young adolescents between the ages of 11 and 13 years in a naturalistic setting (ie, at home, unmonitored). This age range is of particular interest to clinicians and researchers, because it is a transitional stage [13]. This transition from childhood to adolescence—accompanied by increased independence, a new school environment, the onset of puberty, and shifting peer relationships—can be highly stressful and may lead to the emergence of depressive and anxiety symptoms. The influence of stressors on psychopathology in youth is under intensive investigation and conclusions are still difficult to draw [14]. Understanding the etiology of psychiatric disorders is critical to early intervention and most disorders have their onset during adolescence [15-18]. Therefore, surveying adolescents early on in development and following them longitudinally will further our understanding of adolescent development.

Thus, the aim of this study was to examine whether young adolescents can accurately fill out Web-based questionnaires about depression and anxiety, the two most common psychiatric disorders among adolescents (lifetime prevalence of 25% of anxiety disorders and 13% of mood disorders in 13- to 18-year-olds [18]). We accomplish this by comparing Web-based with standard paper-and-pencil versions of depression and anxiety questionnaires.

## Methods

### Sample and Design

Twenty-eight children between the ages of 11 and 13 years (mean 12.78 years and SD 0.78; 18 females, 64%) participated in this study. The study was briefly introduced to 2 classes at a Swiss secondary school. Students who wished to participate sent back their contact information in addition to a consent form signed by their parents or legal guardian and themselves. Once recruited into the study, participants were randomly assigned to complete either the paper-and-pencil version or the Web-based version first in a randomized crossover design. In the paper-and-pencil condition, participants received the questionnaires by mail and a self-addressed stamped envelope was provided for returning the questionnaires. In the Web-based condition, participants received an identification number, a password, and a link to the survey website. Parents were instructed to leave the child alone to fill out the Web-based and paper-and-pencil forms. After completing the first condition, participants received the questionnaires of the alternate condition so that the second assessment was not more than 2 weeks from the first. This rather short lag time was chosen to limit the influence of changes in mental state on the completion of the state-sensitive questionnaires. Participants received compensation in the form of gift vouchers for taking part in the study, which was approved by the Ethics Committee in Bern, Switzerland.

### Questionnaires

The German version (Depressions-Inventar für Kinder und Jugendliche, DIKJ) of the Children’s Depression Inventory (CDI; [19]) was used to measure depressive symptoms. This scale is a well-established self-report measure of depressive symptoms appropriate for children between the ages of 7 and 17 years. This scale consists of 26 items, which are each scored from 0 to 2, and thus the total score of this scale yields values ranging between 0 and 52.

The German version of the Spence Children’s Anxiety Scale (SCAS) was used to measure anxiety symptoms. This scale was designed to assess anxiety symptoms in individuals between the ages of 8 and 15 years and consists of 38 items, which are scored on a 4-point scale—thus scores range between 0 and 114. This scale also permits calculation of subscores, allowing for the evaluation of anxiety across specific domains [20-22].

### Statistical Analysis

Statistical analysis was conducted with SPSS version 23.0.0.0. Intraclass correlation coefficients (ICCs) were used to examine the degree of correspondence between paper and Web-based versions. Intraclass correlation coefficient values range between 0 and 1 and are conventionally categorized as follows: 0-.2 poor, .2-.4 fair, .4-.6 moderate, .6-.8 substantial, and .8-1.0 almost perfect [23]. The Web-based version was considered reliable when an ICC value equal to or greater than .8 (minimum of substantial agreement) was obtained. Additionally, Kendall tau correlations were used to measure the equivalence between the paper-and-pencil and Web-based versions, and Wilcoxon tests were performed to test for statistically significant differences between the two conditions.

## Results

On average, it took 17 minutes (SD 6) to fill out the Web-based questionnaires, which consisted of the SCAS, CDI, and an additional 52 questions about sleep behavior and quality resulting in a total of 116 questions. Because paper-and-pencil questionnaires were filled out at home, no data on time taken to complete the forms were available. The evaluation of the paper-and-pencil and Web-based versions resulted in an overall ICC of .88 (*P*<.001) for the CDI and an ICC of .95 (*P*<.001) for the SCAS (Table 1). According to the criteria of Landis and Koch [23], ICC values higher than .8 fall into the category of “almost perfect” indicating the highest degree of reliability. Kendall tau correlation was also significant with correlation coefficients (*r*) of .58 (*P*<.001) for the CDI and .72 (*P*<.001) for the SCAS. Additionally, Wilcoxon tests comparing paper-and-pencil with the Web-based condition showed no significant difference (Table 1), with the exception of the subscore for Physical Injury Fear of the SCAS.

**Table 1:** The degree of correspondence between the paper-and-pencil and Web-based versions of depression and anxiety questionnaires filled out by 11- to 13-year-old children.

Questionnaire		Paper-and-pencil, mean (SD)	Web-based, mean (SD)	ICC^a^ (*P*)	*Z*^b^ (*P*)	τ^c^ (*P*)
Total SCAS^d^		19.46 (12.64)	21.11 (13.41)	.95 (<.001)	−1.36 (.173)	.72 (<.001)
	Separation Anxiety	2.43 (2.77)	2.75 (2.99)	.92 (<.001)	−0.82 (.410)	.75 (<.001)
	Social Phobia	4.68 (2.94)	4.61 (3.01)	.91 (<.001)	−0.43 (.666)	.66 (<.001)
	Obsessive Compulsivness	4.07 (3.03)	4.07 (2.98)	.92 (<.001)	−1.50 (.881)	.71 (<.001)
	Panic Agoraphobia	2.26 (2.80)	1.78 (2.74)	.90 (<.001)	−1.40 (.163)	.49 (.002)
	Physical Injury Fear	2.32 (2.18)	3.04 (2.78)	.90 (<.001)	−3.06 (.002)	.86 (<.001)
	Generalized Anxiety	4.36 (2.36)	4.82 (2.61)	.86 (<.001)	−1.45 (.148)	.60 (<.001)
Total CDI^e^ (DIKJ^f^)		6.61 (4.46)	7.39 (4.41)	.88 (<.001)	−1.09 (.277)	.58 (<.001)

^a^ICC: intraclass correlation coefficient.

^b^*Z*: Wilcoxon-Mann-Whitney test.

^c^τ: Kendall tau-b.

^d^SCAS: Spence Children’s Anxiety Scale.

^e^CDI: Children's Depression Inventory.

^f^DIKJ: Depressions-Inventar für Kinder und Jugendliche.

## Discussion

In this randomized study, we show that Internet-based data collection of mental health questionnaires is feasible and reliable in early adolescence outside a highly supervised environment. A number of studies have reported feasibility and acceptance of Web-based questionnaires evaluating health among children [24-27], teenagers [11,12,26-29], and adults [30-40]; however, very few studies have examined the reliability of Web-based questionnaires under unsupervised conditions in a sample of young adolescents. For example, Mangunkusumo et al [25] found comparable responses on an Internet versus paper mode of health questionnaires in an elementary school cohort (ages 10-12 years), using a randomized within-subject design. However, the children in this study were intensively supervised by their teachers and changing modalities happened during the same school lesson with only a 5-minute break in between, thus making a memory bias in favor of high correspondence likely. Our within-subject results show a similar outcome in an unsupervised setting and with a longer lag time between assessments, where young adolescents filled out questionnaires during their free time without close supervision—an important modification for the feasibility of longitudinal study designs.

Furthermore, our results not only show high ICC values in the overall sum scores of CDI and SCAS, but they also show high ICC and correlation scores in the subscores of the SCAS. Only the Physical Injury Fear subscale differed between the Web-based and the paper-and-pencil groups and may be due to the small number of items that are included in each subscore.

Additionally, the adolescents in our study were able to answer a relatively large number of items (total of 116 items) in a short time, which shows the feasibility of collecting large datasets. Furthermore, despite our modest sample size, we were able to demonstrate high ICC values, which indicate high intrarater reliability in comparison to interrater variability. Nevertheless, the small sample size is a limitation of our study.

In summary, our study suggests that Internet-based data collection in young adolescents in the field of mental health and beyond is feasible and reliable. Consequently, Internet-based questionnaires can be implemented in larger longitudinal studies in the future in order to develop a better understanding of adolescent behavior and follow the emergence of psychiatric disorders, which can lead to prevention and better treatment.

## Abbreviations

CDI: Children's Depression Inventory
DIKJ: Depressions-Inventar für Kinder und Jugendliche
ICC: Intraclass Correlation Coefficient
SCAS: Spence Children’s Anxiety Scale
